# Declarative Programming with Temporal Constraints, in the Language CG

**DOI:** 10.1155/2015/540854

**Published:** 2015-03-29

**Authors:** Lorina Negreanu

**Affiliations:** POLITEHNICA University of Bucharest, Splaiul Independentei 303, 060042 Bucharest, Romania

## Abstract

Specifying and interpreting temporal constraints are key elements of knowledge representation and reasoning, with applications in temporal databases, agent programming, and ambient intelligence. We present and formally characterize the language CG, which tackles this issue. In CG, users are able to develop time-dependent programs, in a flexible and straightforward manner. Such programs can, in turn, be coupled with evolving environments, thus empowering users to control the environment's evolution. CG relies on a structure for storing temporal information, together with a dedicated query mechanism. Hence, we explore the computational complexity of our query satisfaction problem. We discuss previous implementation attempts of CG and introduce a novel prototype which relies on logic programming. Finally, we address the issue of consistency and correctness of CG program execution, using the Event-B modeling approach.

## 1. Introduction

Specifying and reasoning about phenomena that evolve in time are essential traits of any intelligent system. Their key components are usually identified as (i) representing the temporal behaviour of a system and (ii) extracting information which is otherwise implicit in the system representation. In the traditional line of research, temporal representation and reasoning are deployed for (program) verification [1]. Thus, the entire system behaviour is encoded by some form of labelled transition graph (Kripke Structure), and temporal logic is used for expressing specific properties of the underlying system. Finally, model checking [2] is employed for verifying whether the property is entailed by the system at hand. Unlike the traditional approach, we focus on capturing nonnecessarily deterministic evolutions of a system. Thus, instead of characterizing all possible behaviours, by unfolding, for example, a transition system and examining all paths, we look at a single “*evolution path*.” We consider that our approach has interesting advantages with respect to the traditional line of research based on temporal logics such as LTL, CTL (for a more detailed motivation see, e.g., [3]). We are less interested in eventuality (e.g., fairness constraints) or maintenance (e.g., safety constraints) of properties, which are typical for model checking [4–7] and for deductive reasoning [8–11] in temporal logics. Instead, we would like to identify temporal relations in the occurrence of properties, in the spirit of Allen's Interval Algebra [12]. As an example, consider identifying “*those individuals which were married at least twice*.” This amounts to finding those properties “*married*” which occur one* after* the other and which enrol the same individual.

Our framework consists of (i) a* representation* of an evolution path of a system, one which is specifically tailored for capturing temporal relations between the system properties, (ii) a* temporal language* which we employ for expressing complex temporal constraints between properties, as shown in the above example, and (iii) a* rule-based programming language*, CG, which allows the programmer to specify time-dependent programming. Rule-based programming languages operate on a working memory of factual information: they check rule applicability against the working memory and subsequently modify the latter, by effectively applying the rules. CG follows the same principle; only here the working memory has a* temporal structure*, which is precisely (i). Specifying when a rule is applicable is done using (ii). Applying the rule means executing actions which are aimed at coercing the system evolution according to the programmer's intentions.

CG can be highly effective in specifying intelligent device behaviour in intelligent houses, as illustrated in [13–16]. Also, CG has been employed for temporal data mining [3]; finally, (i) and (ii) were also used as a means for representing game outcomes of multiagent systems [17].

The aim of this paper is to (a) build an all-encompassing view of our approach, (b) present our already established main theoretical results, (c) introduce a novel implementation based on Prolog, and, finally, (d) examine aspects pertaining to* correctness* of our approach. (a) has already been discussed in different variants, in [3, 13–17]; (b) has been the subject of [3, 14, 17]. (c) and (d) however are new contributions which, to our knowledge, have not been considered yet.

The rest of the paper is structured as follows. In Section 2, we introduce the main primitives of our modeling approach. In Section 3, we review the temporal language *L*_*ℋ*_ and its computational properties. In Section 4 we illustrate the rule-based language CG and in Section 5 we examine aspects pertaining to its correctness. In Section 6 we illustrate a lightweight implementation for CG and finally, in Section 7, we conclude.

## 2. Modeling Evolving Applications

Our approach relies on describing the state of the modelled domain as a set of relationships between the actors of the domain, relationships called* qualities* in what follows: quality relation instances of the form *R*(*i*_1_,…, *i*_*n*_) where *R* designates the property at hand, *n* is the arity of *R*, and *i*_1_,…, *i*_*n*_ are the individuals enrolled in the relationship. For instance, the quality* Married(John,Alice)* designates a binary relationship between two individuals, while* On(ac)* designates a property of the device* ac* (air conditioner).

A state, as seen in the conventional approach, is unpacked into a set of qualities, which portrays the status of the domain over a finite time interval, given that no changes are present during the interval at hand. A state transition corresponds to a change in the domain: the commencement of new qualities or the termination of existing ones. Such a change is triggered by* actions*. An action is also an instance of the form *a*(*i*_1_,…, *i*_*n*_), which designates an instantaneous event of type *a*, which enrols individuals *i*_1_,…, *i*_*n*_. For instance,* Marries(John,Alice)* is an action which changes the status of John and Alice: having been initially single, they now become married. Similarly,* TurnOn(ac)* is an action which changes the status of the air conditioner.

**Figure 1:**
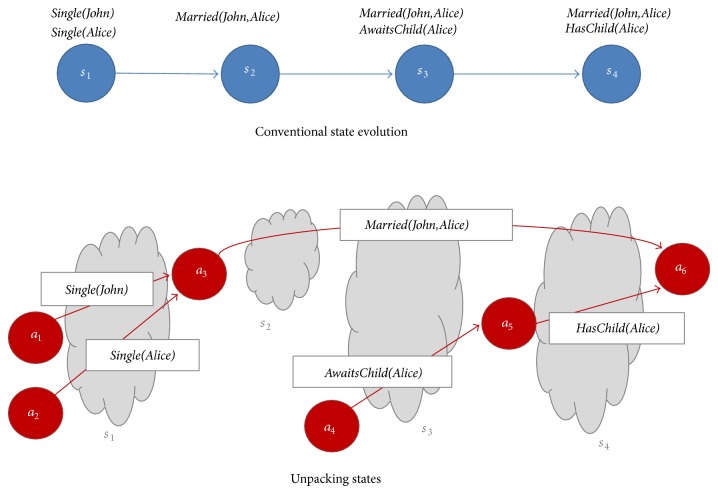
Unpacking states.

State unpacking is illustrated in Figure 1. Above, a conventional transition system is used to describe the evolution of a domain: John and Alice are initially single, they become married, and Alice awaits a child that also comes later on. Below, we use a quality-oriented description: the focus shifts from states labelled with certain properties to qualities introduced and terminated by actions. In the former approach, the lifespan of properties is implicit: one must examine the sequence of states on which the property continuously holds. For example,* Married(John,Alice)* holds from *s*_2_ to *s*_4_. In our approach, the lifespan of qualities is represented explicitly, by their initiating and terminating actions. For instance,* Married(John,Alice)* holds from the moment *a*_3_ was executed until *a*_6_ was executed. We assume *a*_3_ designates the marriage action while *a*_6_ is a special action belonging to the current moment. We have not labelled actions to avoid cluttering the figure.

It is easy to see that the two description styles are equivalent. Nevertheless, we argue that our quality-oriented description suits better applications where the timing is important and, moreover, where the temporal relationship between qualities is an essential issue. Also, by avoiding unnecessary relabellings of sequences of states, we obtain a more compact representation which speeds up processing and saves space.

### 2.1. Domain Representation

In what follows, we distinguish between an ontological representation of a domain itself and a temporal one. The former is* temporally flat* and provides the taxonomy which characterizes the domain. The latter is, in essence, a temporal structure which instantiates the taxonomy, as we will further show.

#### 2.1.1. Individuals

The actors of a described domain are* individuals*. They are atomic, unique, and identifiable by themselves. They are used to represent entities from the domain (John and Alice or the air conditioner, in the above examples), as well as primitive values of use in the language (e.g.,* 20 degrees*, the time-stamp* 18:50:00*, etc.) or even the environment seen as an entity in itself. Seen from the programming perspective, individuals behave much like atoms in the language of Prolog: they are string literals without an explicit type.

#### 2.1.2. Actions

An action corresponds to an instantaneous stimulus applied to one or more individuals. Actions are represented as relation instances *a*(*i*_1_,…, *i*_*n*_) where *a* designates the action type, *n* is the arity of *a*, and *i*_1_,…, *i*_*n*_ are the individuals that the action enrols.

#### 2.1.3. Qualities

A quality designates a time-dependent property *P*(*i*) of individual *i* or an n-ary relationship *R*(*i*_1_,…, *i*_*n*_), between individuals *i*_1_,…, *i*_*n*_.

#### 2.1.4. Time

Individuals, actions, and qualities are merely taxonomical entities. In what follows, we add temporal dimension to each one. First, we consider individuals as perennial. Their existence is unaltered by the evolution of the domain. The temporal dimension of an action is an* action node*. A group of action nodes uniquely identify a moment of time when they occur, provided that their occurrence is simultaneous. We call a collection of such action nodes a* hypernode*. The temporal dimension of a quality *q* = *R*(*i*_1_,…, *i*_*n*_) is a* quality edge *(*a*, *b*) which spans action nodes *a* and *b*. *a* models the event which has initiated the enrolment of (*i*_1_,…, *i*_*n*_) in *R*, while *b* models the event responsible for its termination. The lifespan of *q* is given by the temporal moments when *a* and *b* occur, respectively.

These temporal components are glued together in a structure called temporal graph (short t-graph).

**Definition 1 (temporal graph).**

A temporal graph is an oriented graph *ℋ* = (*A*, *E*), where *A* designates the set of action nodes and *E* that of quality edges, together with a partition *H* over *A*. One denotes the elements *h*_*i*_ ∈ *H* as hypernodes. One assumes elements of *A* and *E* have a unique label of the form *R*(*i*_1_,…, *i*_*n*_), which one denotes by *ℒ*(*a*) for *a* ∈ *A* and *ℒ*(*a*, *b*) for (*a*, *b*) ∈ *E*, respectively. For a more rigorous treatment, one refers the reader to [3].

**Figure 2:**
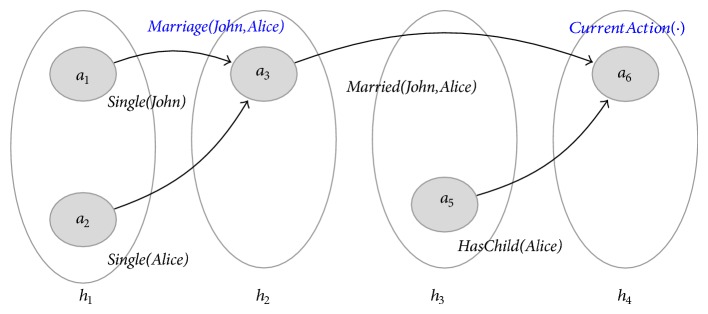
The temporal graph *ℋ*_1_ describing John and Alice's evolution.

The domain evolution described in Figure 1 is captured by the t-graph from Figure 2 (we have omitted the representation of the quality* AwaitsChild(Alice)*, due to limited space). We have represented action labels in blue. Also, in order to make the figure more legible, we have only labelled those actions subject to our discussion.

**Definition 2 (temporal ordering, precedence).**

**Figure 3:**
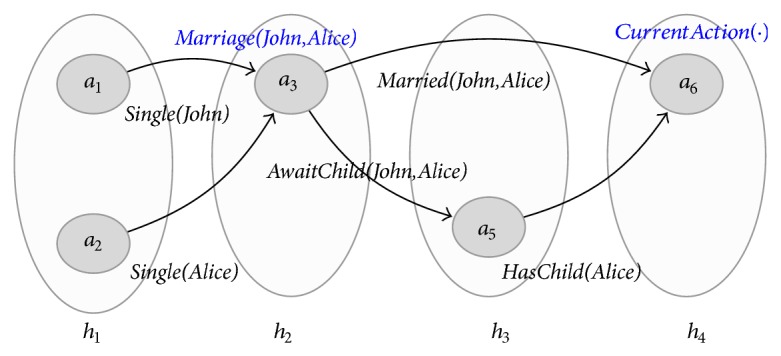
The temporal graph *ℋ*_1_′ describing a more precise evolution of John and Alice.

A hypernode *h immediately precedes* another (*h*′) in a t-graph, if and only if there exists a quality edge (*a*, *b*) such that *a* ∈ *h* and *b* ∈ *h*′. Immediate precedence is a partial ordering of hypernodes, as illustrated in Figure 2. For instance, *h*_1_ immediately precedes *h*_2_ and *h*_3_ immediately precedes *h*_4_; however the same cannot be said about *h*_2_ and *h*_3_. Although represented in sequence in Figure 2, *h*_2_ and *h*_3_ need not occur in this particular order. Thus, it might be the case that Alice has a child prior to the marriage to John or that the child comes after the marriage. Such information is absent from *ℋ*_1_ and neither conclusion could be made. However, in Figure 3, the ambiguity is lifted by the presence of the quality (*a*_3_, *a*_5_), labelled* AwaitChild(John,Alice)*.

We denote by ≻ the transitive closure of the immediate precedence relationship, described previously. If *h*≻*h*′, we say *h* precedes *h*′.

An action node *a* ∈ *h* (immediately) precedes *a*′ ∈ *h*′ if and only if *h*≻*h*′.

Let *q* = (*a*, *b*) and *q*′ = (*a*′, *b*′) be two quality edges. *q* occurs* before* (after) *q*′ if and only if *b* precedes *a*′ (*b*′ precedes *a*); *q* occurs* just before* (just after) *q*′ if and only if *a* precedes *a*′ and *a*′ precedes *b* (*a*′ precedes *a* and *a* precedes *b*′); *q* overlaps with *q*′ if and only if *a*, *a*′ and *b*, *b*′ are simultaneous, respectively; *q* meets *q*′ if and only if *b*, *a*′ are simultaneous or coincide; *q* contains *q*′ if and only if *a* precedes *a*′ and *b* precedes *b*′. The relationships between quality edges are inspired from Allen's Interval Algebra [12].

For instance, in Figure 2, (*a*_1_, *a*_3_) meets with (*a*_3_, *a*_6_). Similarly, in Figure 3, (*a*_1_, *a*_3_) is before (*a*_5_, *a*_6_). The same does not hold in Figure 2.

## 3. Asking Temporal Questions: Queries

### 3.1. The Language *L*_*ℋ*_

Temporal graphs store time-dependent information. They act as a temporal knowledge base for an ever changing domain. In what follows, we present a means for interrogating the knowledge base, the language *L*_*ℋ*_.

Consider a possible query such as* Married(John,Alice)*. Intuitively, the question intended here is whether John is married to Alice. Judged with respect to time, the question becomes as follows: “*Is it the case that John was married to Alice, at any point in the evolution of the domain?*” The answer to such a query formulated with respect to a temporal graph *ℋ* will return all the quality edges which satisfy it, that is, all quality edges (*a*, *b*) from *ℋ* such that *ℒ*(*a*, *b*) = *Married*(*John*, *Alice*).

Next, consider the query* Married(X,Alice)*. Here, *X* is a variable. We use the Prolog-style convention and denote variables by capitals. The query encodes the following question: “*Was Alice ever married to someone?*” The answer will produce a possibly empty set of* records*. If the set is nonempty, then each* record* is a (different) witness that the answer to the above question is* yes*. In our case, each record will contain a substitution *X* = *i* (e.g., *X* = *John*) as well as the quality edge (*a*, *b*) such that *ℒ*(*a*, *b*) = *Married*(*i*, *Alice*).

Further on, consider the query* Married(X,Y) after Married(Y,John)*. The query will identify all marriages of some individual *X* to *Y* which precede those of *Y* to John. In this case, each record will store the individual values for *X* and *Y*, together with the quality edges labelled accordingly.

Also, we have

- ‖*Single*(*X*)‖_*ℋ*_1__ = {{(*a*_1_, *a*_3_)}, {(*a*_2_, *a*_3_)}};
- ‖*Single*(*X*)*before* *hasChild*(*X*)‖_*ℋ*_1_′_ = {{(*a*_2_, *a*_3_), (*a*_5_, *a*_6_)}};
- ‖*Single*(*X*)*before* *hasChild*(*X*)‖_*ℋ*_1__ = *∅*;
- ‖*Single*(*X*)*before* *hasChild*(*X*)∧*Single*(*X*)*meets* *Married*(*X*, *John*)‖_*ℋ*_1_′_ = {{(*a*_2_, *a*_3_), (*a*_3_, *a*_6_), (*a*_5_, *a*_6_)}}.

The evaluation of query (a) (in *ℋ*_1_) will produce two records, each containing the quality edge which satisfies the label. The evaluation of query (b) (in *ℋ*_1_′) will produce one record of two qualities, one for each label which occurs in the formula. The evaluation of query (c) will produce no record, while that of query (d) will produce one record of three qualities. Each record implicitly contains a mapping function between each satisfied label and its corresponding quality. Since such a function is not vital for the discussion of our approach in this paper, we have chosen to omit it.

**Definition 3 (the language *L*_*ℋ*_).**

Let *𝕍*ars designate a set of variables. A term, denoted by *t*, is either a variable or an individual. The syntax of *L*_*ℋ*_ is recursively defined as follows:

(1) φ::=R(t1,…,tn) ∣ hhhhR(t1,…,tn)∝φ1 ∣ hhhh¬φ1 ∣ hhhh⋀iR(t1,…,tn)∝φi,

where ∝ designates any temporal precedence relations between quality edges specified in Definition 2, *R* is some quality type of arity *n*, and *t*_1_,…, *t*_*n*_ are terms.

We denote by ‖*φ*‖_*ℋ*_ the set of records which satisfy the query *φ* in the temporal graph *ℋ*. ‖·‖_*ℋ*_ constitutes the semantics of *L*_*ℋ*_, which we will not discuss in detail. Instead, we refer the reader to [3].

Negation and conjunction require some clarifications. The formula ¬*φ*, interpreted in a t-graph *ℋ*, should be interpreted as *φ is not true in ℋ*; hence ‖*φ*‖_*ℋ*_ = *∅*. Hence, a (sub)formula of the type ¬*φ* will not generate a record, when satisfied.

Conjunction is used to express multiple temporal constraints over the same quality. For instance, the formula* Single(X) before hasChild(X) *∧* Single(X) meets Married(X,John)* expresses two constraints on the quality* Single(X)*, which must be simultaneously satisfied by each quality edge from the record of* Single(X)*.

### 3.2. *L*_*ℋ*_ Complexity

**Proposition 4 (see [3, 18]).**

Let *ℋ* be a t-graph and *φ* a formula of *L*_*ℋ*_. Checking ‖*φ*‖_*ℋ*_ ≠ *∅* is NP-complete.

*Sketch.* We prove hardness only. For membership, see [3, 18]. As a reduction, we use the conjunctive query problem [19]: given a structure *S* and a sentence of the form

(2) $$\begin{array}{c} \varphi_{c}=\exists x_{1}\cdots \exists x_{n}C_{1}\wedge \cdots \wedge C_{n} \end{array}$$

where each *C*_*i*_ is an atomic formula containing no free variables, the problem asks if *S* makes the formula true. From *φ*_*c*_ we build an *L*_*ℋ*_ formula as follows: for each *C*_*i*_ we build the *L*_*ℋ*_ formula (gadget): *C*_*i*_ *overlaps* *Fix*(*e*). *φ* is the conjunction of such gadgets. Next, from the structure *S*, we build a t-graph *ℋ*: (i) we create a quality edge *q* = (*a*, *b*) labelled* Fix(e)*; (ii) for each relation instance $C_{i}(\bar{c})$ in *S*, we build the quality edge *q*_*i*_ = (*a*_*i*_, *b*_*i*_) labelled $C_{i}(\bar{c})$, such that *q*_*i*_ overlaps with *q*.

(⇒). Assume *S* makes *φ*_*c*_ true. In particular, *S* makes ∃*x*_1_ ⋯ ∃*x*_*n*_. *C*_*i*_ true, for each *C*_*i*_. Hence, there exists a quality edge in *ℋ* which satisfies the query *C*_*i*_ *overlaps* *Fix*(*e*), for each *C*_*i*_. Thus, ‖*φ*‖_*ℋ*_ is nonempty.

(⇐). Assume ‖*φ*‖_*ℋ*_ is nonempty and let *r* be some record of ‖*φ*‖_*ℋ*_. *r* must contain, for each *L*_*ℋ*_ subformula *C*_*i*_ *overlaps* *Fix*(*e*), a quality edge (*a*_*i*_, *b*_*i*_) which satisfies it; hence one labelled $C_{i}(\bar{c_{i}})$, which is also a relation instance of *S*. Therefore, for all *C*_*i*_, $\bar{c_{i}}$ are evidence that the conjunctive query *φ*_*c*_ is true in *S*.

The computational complexity of the query satisfaction problem may seem discouraging at first sight. However, the source of complexity can be found in the maximal arity of the underlying qualities. For instance, given a formula Q(X,Y,Z), substitution would require building *n* ^3^ possible labels Q(i,j,k), where *n* is the total number of individuals. For formulae where the arity is an unbounded *m*, the possible labels become exponential: *n* ^*m*^. However, in practice, it is less likely that queries will be formulated with qualities of arity larger than 4. Thus, under this assumption, the computational complexity of satisfying queries becomes manageable.

## 4. Temporal Inference: CG

### 4.1. Updating Temporal Graphs

As illustrated up to this point, the language *L*_*ℋ*_ is a means for investigating the evolution of a domain described as a temporal graph. The latter acts as a structured log and offers no means of interfering with the domain's current and future evolution. In this section we make a step forward and describe a means of achieving this. We introduce yet another language, which we call CG, which can be used to make changes to a domain. Unlike *L*_*ℋ*_, CG is not a logical/temporal language, but a* programming language*, operating on a knowledge base which constitutes a temporal graph. The basic programming unit of CG is the* rule*. A rule consists of (i) a set of preconditions, (ii) an action, and (iii) a set of effects. An example is given below (in what follows, we abandon our “John and Alice” example theme for a more practical one, related to the field of application of CG, namely, that of agent programming):

 
*rule* r1:
 
On(X) as q
 
OperatesUnderCG(X)
 
=> turnOff(X) as a =>
 
terminate q in a, create Off(X) from a.

Each precondition is given as an *L*_*ℋ*_-formula, where qualities can be named for later use (e.g., On(X) as q). The action (ii) specifies a stimulus under which the rule-at-hand is activated. In our example, turnOff is the respective action. Once a rule is activated, each precondition must be evaluated, in order to establish whether the rule should be applied or not. Let *φ*_1_ ⋯ *φ*_*n*_ be the preconditions of a rule. By evaluating *φ*_1_ we obtain the set ‖*φ*_1_‖_*ℋ*_, which contains a list of records. Each such record *r* will contain the qualities which have satisfied *φ*_1_, together with a substitution for each variable occurring in *φ*_1_. The evaluation of the next precondition (*φ*_2_) will be achieved with respect to the substitution in each *r*. When evaluating the last precondition with respect to all previous records, we will obtain complete substitutions of all variables occurring in the preconditions. Each such substitution, together with the matched qualities, is an* activation record*.

If at least one activation record exists for a rule, we say it is applicable, which means the effects (iii) can be enforced on the temporal graph. The effects of a rule consist in adding new qualities to the temporal graph or terminating existing ones. Both initiation and termination are relative to existing qualities from the temporal graph and to the current moment. For instance, terminate q in a will have the effect of terminating the matched quality q, in the action node corresponding to a. Similarly, create Off(x) from a will create a new quality edge labelled Off(x), which spans *a* and a special* current action*, belonging to the current moment of time and which implicitly terminates all qualities which are known to hold at the current moment.

Rules such as r1 model ontological knowledge. They maintain temporal graphs by making implicit information explicit. In the previous example, the occurrence of a signal turnOff(a) will produce the disconnection of the device a, provided that it is controlled by the application (OperatesUnderCG(a) is true). The rule explicitly states this, by adding the Off(a) quality, starting from the exact time when turnOff(a) is executed. Such rules are* reactive to actions only*.

We also allow programmers to execute their own actions and thus steer the evolution of the domain in the desired way. For instance, the very simple rule

 
*rule* r2:
 
On(X),AirConditioner(X),Open(win)
 
=> turnOff(X)

will turn off all air conditioners, whenever the window is opened.

In what follows we provide a grammar for the language CG:

 
<rule>::= rule <id>: <pl> [=>  <act>]
 
=>  <el>
 
<pl>:: =*φ* [,<pl>]
 
<act>,<qual>::= *R*(*t*_1_,…, *t*_*n*_)
 
<el>::= <eff> [,<el>]
 
<eff>::= <act>
      
 ∣  create  <qual>  from  <id>
      
 ∣  terminate  <id>  in  <id>.

We have denoted by pl and el *precondition list* and* effect list*. Act and qual designate the* action* and* quality* tokens, while eff designates an effect. id is a program identifier used to denominate rules, matched qualities, and/or actions.

## 5. Checking the Correctness of CG Programs

Rule-based programs are usually validated by submitting some sample results to human experts. While it can be helpful it obviously does not provide enough coverage. A formal specification provides an independent standard of accuracy that can be used to check the program output. Our goal is to develop a formal specification for CG programs. We have avoided defining new action logics based on *L*_*ℋ*_—in the spirit of PDI (propositional dynamic logics) [20] or situation calculus [21]—for specifying rules, their preconditions, and effects and opted for existing methods. To this end we will use Event-B specification method [22] and the Rodin platform [23].

A rule-based program has two components: a database of rules and a rule interpreter. The correctness of the rule-based program involves the correctness of the database and the correctness of the interpreter. A correct database of rules is a database where the rules do not contradict. A correct rule interpreter infers all the pertinent conclusions entailed by its facts and rules and does not infer any conclusions that are not justified by them. In order to fit into the Event-B modeling framework, in this section, we have opted for viewing preconditions, actions, and effects as* facts*, thus ignoring the differences between them. This abstraction does not affect the generality of our results and merely serves to make our model more legible.

### 5.1. Event-B Modeling of Facts and Rules

We model the facts by the abstract set FACT. Rules associate a set of facts—the premises—with another fact—the conclusion. If the premises all hold, the conclusion will also hold. In our model we represent the rule database as a relation from the set of facts to facts:

(3) $$\begin{array}{c} rules\in P\left(\text{FACT}\right)⟷\text{FACT}. \end{array}$$

New facts are generated by examining the whole set of facts, applying all the applicable rules, and adding all the new facts to the set. The whole process is repeated until no new facts appear. We model this process as an application of the function* infer*:

(4) $$\begin{array}{c} infer\in P\left(\text{FACT}\right)⟷P\left(\text{FACT}\right) \end{array}$$

having as domain the initial set of facts and codomain the final set of facts. While the initial set of facts also appears in the final set, *infer* may add new facts:

(5) infer=λfacts·facts∈PFACT ∣ facts ∪rulesPfacts.

The expression *rules*[*𝒫*(*facts*)] is the set of all the conclusions of all the rules whose premises match some combination of the initial set of facts, where *𝒫*(*facts*) is the set of all combinations of the initial facts. The expression rules [*𝒫*(*facts*)] use the relational image brackets [·] in order to get the set of conclusions of all the pairs in the relation rules whose premises appear in the set *𝒫*(*facts*).

The function *infer* should be applied until no more conclusions can be inferred. We model the repetitive application of *infer* by the function *closure*, the transitive closure of *infer*, defined by the following axioms:

(6) closure(infer)∈P(FACT)⟶P(FACT)infer⊆closure(infer)closure(infer); infer⊆closure(infer)∀p·infer⊆p∧p; infer⊆p⟹closure(infer)⊆p

that specify the characteristic properties of the irreflexive transitive closure. Given a relation *r* from a set *S* to itself, the irreflexive transitive closure of *r*, denoted by *closure*(*r*), is also a relation from *S* to *S*. The characteristic properties of *closure*(*r*) are as follows:

- relation *r* is included in *closure*(*r*);
- the forward composition of *closure*(*r*) with *r* is included in *closure*(*r*);
- relation *closure*(*r*) is the smallest relation dealing with (i) and (ii).

### 5.2. Rule Consistency

We assume that rules are consistent if, starting from a consistent set of facts, there is no way to infer inconsistent facts. In order to model the inconsistency of facts we use the set *inconsistent* containing mutually exclusive categories, that is, sets of facts that we know are inconsistent:

(7) $$\begin{array}{c} inconsistent\in P\left(P\left(\text{FACT}\right)\right). \end{array}$$

A consistent set of facts contains no more than one element from each set of mutually exclusive categories:

(8) consistent∈P(P(FACT))∀facts,mutually_exclusive·facts∈consistent∧mutually_exclusive∈inconsistent⟹card(mutually_exclusive∩facts)<2,

where *facts* ranges over all sets of consistent facts and *mutually*_*exclusive* ranges over all sets of inconsistent facts.

### 5.3. Specification of Rule-Based Programs

A rule-based program infers conclusions relevant to some goal based on some data. We model the inference process by the event *Inference* (see Specification 1).

**Specification 1: Specification of the inference process.**
*Inference *
 ANY
  data
  goals
 WHERE
  grd1:  data *∈𝒫*(FACT)
  grd2:  goals *∈𝒫*(FACT)
 THEN
  act1:
  
*facts* : ∣*facts*′ ∈ *𝒫*(FACT)∧*facts*⊆*facts*′∧*facts*′⊆(*closure*(*infer*))(*facts* ∪ data)∧conclusions⊆*facts*′
  act2:  conclusions = goals∩(*closure*(*infer*))(*facts* ∪ data)
 END

The variables *facts* and *facts*′ represent the state of the system before and after the execution of the *Inference* event. The nondeterministic assignment operator :∣ expresses that a modification is possible using a before-after-predicate (expressed in the above specification immediately after the first occurrence of :∣, from the act1 action). The data are the new facts that are introduced, and conclusions are the facts that are inferred. The expression (*closure*(*infer*))(*facts* ∪ data) denotes the set of valid facts that can be inferred from the initial set of facts and the new data; *facts*⊆*facts*′ models the assumption that the program never retracts any conclusions; *facts*′⊆(*closure*(*infer*))(*facts* ∪ data) expresses that all new facts are valid inferences; conclusions⊆*facts*′ models the inference of valid goals.

The complete specification is shown in Specification 2.

**Specification 2: The complete specification.**
CONTEXT
Rules_c0
SETS
 FACT
CONSTANTS
 rules
 infer
 closure
 consistent
 inconsistent
AXIOMS
 axm1:  *rules* ∈ *𝒫*(FACT)↔FACT
 axm2:   *infer* ∈ *𝒫*(FACT) → *𝒫*(FACT)
 axm3:   *infer* = (*λfacts* · *facts* ∈ *𝒫*(FACT)∣*facts* ∪ *rules*[*𝒫*(*facts*)])
 axm4:  *closure*(*infer*) ∈ *𝒫*(FACT) → *𝒫*(FACT)
 axm5:   *infer*⊆*closure*(*infer*)
 axm6:  *closure*(*infer*); *infer*⊆*closure*(*infer*)
 axm7:  ∀*p* · *infer*⊆*p*∧*p*; *infer*⊆*p*⟹*closure*(*infer*)⊆*p*
 axm8:  *consistent* ∈ *𝒫*(*𝒫*(FACT))
 axm9:  *inconsistent* ∈ *𝒫*(*𝒫*(FACT))
 axm10:
 ∀*facts*, *mutually*_*exclusive* · *facts* ∈ *consistent*∧*mutually*_*exclusive* ∈ *inconsistent* → *card*(*mutually*_*exclusive*∩*facts*) < 2
 axm11:   ∀*facts* · *facts* ∈ *consistent*⟹(*closure*(*infer*))(*facts*) ∈ *consistent*
END
MACHINE
 Rules0
SEES
 Rules_c0
VARIABLES
 facts
 conclusions
INVARIANTS
 inv1:  *facts* ∈ *𝒫*(FACT)
 inv2:  conclusions ∈*𝒫*(FACT)
EVENTS
 INITIALISATION:
  THEN
   act1:  *facts* = ∅
   act2:  conclusions = ∅
  END
 Inference:
  ANY
   data
   goals
  WHERE
   grd1:  data ∈*𝒫*(FACT)
   grd2:  goals ∈*𝒫*(FACT)
  THEN
   act1:  *facts* : ∣*facts*′ ∈ *𝒫*(FACT)∧*facts*⊆*facts*′∧*facts*′⊆(*closure*(*infer*))(*facts* ∪ data)∧conclusions⊆*facts*′
   act2:  conclusions = goals∩(*closure*(*infer*))(*facts* ∪ *data*)
  END
END

Our model is an abstract one that can be further refined by refining the *Inference* event, by explicitly specifying control methods (e.g., backward/forward chaining), which explicitly specify how new facts are deduced.

### 5.4. Model Validation

The model has been specified and validated using Rodin, an Eclipse-based IDE for Event-B that provides support for refinement and mathematical proofs [23]. The model is validated by discharging proof obligations. The state of development is described in Table 1 with the required proof obligations.

## 6. Implementation

Our previous implementation efforts were either (i) driven by the application context [15, 16, 24, 25] or (ii) attempted to follow closely the algorithm description, in order to highlight correctness [3, 14, 18]. There are lessons to be learned from either approach. For instance, approaches such as [16, 25] are highly dependent on the web service (WS) architecture which is vital for communicating with intelligent devices. Although proficient for the envisaged scenario, (i) lacks portability as well as scalability. While the WS approach has its well-known advantages [26], the author believes WS development can occasionally be hardened by the platform, IDE, and other application constraints. On the other hand, approaches such as [18] which is split between two implementations in two different languages (Haskell/Frege [27] and CLIPS [28]) and is not application-dependent may lack usability. We believe (ii) to be highly dependent on the implementations of the two languages (the former, Frege, a rather experimental language, is known to have unintuitive dissimilarities from Haskell).

Thus, we take a step back and opt for a new approach, one which preserves the application independence of (ii) but which is more user-friendly, easy to use, and reliant on a unique programming environment. The reader may have noticed some similarities between Prolog and CG. One common feature is the query (/clause) matching process which, essentially, is the same for both languages.

Actually, CG can be seen as a temporal layer on top Prolog: the flat knowledge base is replaced by a temporal one (a temporal graph). Each CG rule can be seen as a collection of Prolog clauses. Goal (re)satisfaction corresponds to rule execution for each found activation record.

In order to represent temporal graphs in Prolog, we use the following 5 metapredicates:

     
 node(A).
     
 edge(A,B).
     
 in(H,A).
     
 quality(A,B,q).
     
 action(A,a).

The factual knowledge node(A) indicates that A is an action node, while action(A,a) assigns the label a to A. Similarly, edge(A,B). indicates that (*A*, *B*) is a quality edge, while quality(A,B,q) assigns the label q to (*A*, *B*). Both a and q are arbitrary Prolog predicates. Finally, in(H,A) indicates that H is the hypernode to which action node A belongs. Hypernodes are not prespecified by another predicates, since this would be superfluous. Thus, the set of hypernodes can be identified as the entities H satisfying the goal in(H,_).

The challenge when transforming a CG rule to a set of Prolog sequences is expressing temporal constraints between qualities. To achieve this, we compute the transitive closure of the appropriate direct precedence relation, introduced in Definition 2.

We illustrate this by a simple example, given by the following query:

      
 q(X,Y)  before r(X).

Such a query is transformed into a clause of the following form:

 
find(A,B):-  quality(A,B,q(X,_)),
        
 quality(C,_,r(X)),
        
 precedes(B,C).

Thus, in order to identify the quality edge(s) satisfying the above query, one must find a quality edge labelled r(X) whose initiating action node C must be preceded by B. Precedence is computed as the transitive closure of the edge and simultaneous relations:

 
precedes(X,Y):-  edge(X,Y),  !.
 
precedes(X,Y):-  simultaneous(X,Y),  !.
 
precedes(X,Y):-  edge(X,Z),
          
 precedes(Z,Y),  !.
 
precedes(X,Y):-  simultaneous(X,Z),
          
 precedes(Z,Y),  !.

Note that we have used !(cuts), in order to avoid unnecessary explorations of the knowledge base, once precedence has been established. The simultaneous clause is defined as follows:

 
simultaneous(X,Y):-  in(H,X),
            
 in(H,Y),
            
 not(X = Y),  !.

And it is satisfied if the two action nodes X and Y belong to the same hypernode.

Specifying more complicated queries is achieved compositionally, following the scheme presented above. Executing the effects of a rule reduces to enriching the metarelations defined at the beginning of this section, in a* transactional* manner, in the spirit of [14]. This means, in short, that all effects resulting from rules which are applicable at the same moment of time are added to the knowledge base in a manner which is perceived as simultaneous by the programmer. This implies that the effects of one rule cannot invalidate another, if both rules have been applicable at the same moment.

## 7. Conclusion

Temporal graphs coupled with *L*_*ℋ*_ in CG are a powerful method for performing temporal reasoning and for enforcing time-dependent behaviour within intelligent systems. One major advantage of CG is that time is not encoded explicitly in other program-dependent structures. Time is a language primitive in itself, and this design choice makes program development straightforward, even for the inexperienced programmer. Besides being easy to read, declarative programs can also be easy to verify, as illustrated in Section 5. The prototype described in Section 6 relies on the cost-expensive resolution of Prolog; however more efficient implementations are possible. We leave such an endeavour for future work.

## Figures and Tables

**Table 1:** 

Name	Total	Auto	Manual	Reviewed	Undischarged
Rules	9	6	3	0	0
Rules_c0	6	4	2	0	0
Rules0	3	2	1	0	0
